# Memristor Circuits for Simulating Neuron Spiking and Burst Phenomena

**DOI:** 10.3389/fnins.2021.681035

**Published:** 2021-06-10

**Authors:** Giacomo Innocenti, Mauro Di Marco, Alberto Tesi, Mauro Forti

**Affiliations:** ^1^Dipartimento di Ingegneria dell'Informazione, Università degli Studi di Firenze, Firenze, Italy; ^2^Dipartimento di Ingegneria dell'Informazione e Scienze Matematiche, Università degli Studi di Siena, Siena, Italy

**Keywords:** neuron, spiking, bursting, memristor, pulse-programmed circuit, harmonic balance

## Abstract

Since the introduction of memristors, it has been widely recognized that they can be successfully employed as synapses in neuromorphic circuits. This paper focuses on showing that memristor circuits can be also used for mimicking some features of the dynamics exhibited by neurons in response to an external stimulus. The proposed approach relies on exploiting multistability of memristor circuits, i.e., the coexistence of infinitely many attractors, and employing a suitable pulse-programmed input for switching among the different attractors. Specifically, it is first shown that a circuit composed of a resistor, an inductor, a capacitor and an ideal charge-controlled memristor displays infinitely many stable equilibrium points and limit cycles, each one pertaining to a planar invariant manifold. Moreover, each limit cycle is approximated via a first-order periodic approximation analytically obtained via the Describing Function (DF) method, a well-known technique in the Harmonic Balance (HB) context. Then, it is shown that the memristor charge is capable to mimic some simplified models of the neuron response when an external independent pulse-programmed current source is introduced in the circuit. The memristor charge behavior is generated via the concatenation of convergent and oscillatory behaviors which are obtained by switching between equilibrium points and limit cycles via a properly designed pulse timing of the current source. The design procedure takes also into account some relationships between the pulse features and the circuit parameters which are derived exploiting the analytic approximation of the limit cycles obtained via the DF method.

## 1. Introduction

The ever-growing need of computing power to handle data intensive applications is seriously challenging conventional digital von Neumann computing architectures (Bonomi et al., 2012; Satyanarayanan, 2017; Williams, 2017). The physical separation between the computing and memory units can indeed generate long latency time and large energy consumption when data intensive tasks are performed. In this context, researchers look at the emerging nanoscale analog devices, such as memristors, as a viable approach for developing new computing paradigms, based on in-memory and analog computation, which are potentially capable to overcome the limitations of conventional computer architectures (Waldrop, 2016; Zidan et al., 2018; Krestinskaya et al., 2020).

The memristor (a shorthand for memory resistor) is the fourth basic passive circuit element theoretically introduced by Prof. Leon Chua in 1971 (Chua, 1971). The appealing features of a memristor are *non-volatility*, i.e., the memristor capability to hold data in memory without the need of a power supply, and *non-linearity*, which makes memristor circuits capable to generate quite a rich variety of oscillatory and complex dynamics. The combination of these two features enables in-memory computing, i.e., the co-location of computation and memory in the same device (Ielmini and Wong, 2018). In-memory computing, which resembles a basic principle of brain computation, can provide several advantages, such as low energy consumption, high bandwidths, excellent scalability, thus lending itself as quite a promising novel computing approach in the field of artificial intelligence and big data (Ielmini and Pedretti, 2020). Memristor circuits have been already positively used to address several tasks, including the solution of global optimization, constraint satisfaction and linear algebra problems (Yang et al., 2013; Wang et al., 2015; Kumar et al., 2017). They are also used as building blocks in reservoir computing systems (Du et al., 2017) and neuromorphic computing for on-line signal processing tasks (Di Marco et al., 2016, 2017; Di Marco et al., 2017; Ascoli et al., 2020a,b).

Since the very beginning it was clear that understanding the peculiar dynamical features of memristor circuits is the key step for developing analog in-memory computing schemes. It has been definitely shown that memristor circuits are capable to generate quite a large variety of dynamical behaviors (Corinto et al., 2011; Corinto et al., 2019; Pershin and Di Ventra, 2011; Xu et al., 2016; Yuan et al., 2016; Di Marco et al., 2018), including bursting oscillations (see e.g., Wang et al., 2019 and references therein). Recently, a new technique, named flux-charge analysis method (FCAM), has been introduced for the analysis of memristor circuits in the flux-charge domain (Corinto and Forti, 2016, 2017, 2018). FCAM permits to show that the dynamical richness displayed by memristor circuits is a consequence of the property that the state space of any given circuit, i.e., with its parameter having fixed values, contains infinitely many invariants manifolds (foliation property of the state space). This specific property implies that in memristor circuits there is the coexistence of infinitely many different attractors, a property referred to as *multistability*. Moreover, structural changes of the attractors are observed when the initial conditions are varied while keeping constant the values of the circuit parameters, a peculiar phenomenon which is referred to as bifurcations without parameters (Corinto and Forti, 2017; Di Marco et al., 2018; Innocenti et al., 2019b). In particular, Di Marco et al. (2018), Innocenti et al. (2019b) have employed techniques within the Harmonic Balance (HB) context for predicting limit cycles and their bifurcations by first showing that the dynamics of the memristor circuit admits an equivalent input-output representation, which has been recently extended also to circuits containing memory elements (Innocenti et al., 2020).

Among other applications, it has been soon recognized that memristors can be successfully employed as synapses in neuromorphic circuits (see e.g., Jo et al., 2010; Adhikari et al., 2012; Thomas, 2013; Kim et al., 2015; Hu et al., 2016; Hong et al., 2019). It has been also pointed out that memristor circuits appear to be suited for modeling some features of the dynamics of neurons. Some contributions provide a memristor representation of popular neuron models (see e.g., Chua et al., 2012; Usha and Subha, 2019 for the Hodgkin-Huxley axon), while others show how to mimic some typical dynamics displayed by cortical neurons (see e.g., Babacan et al., 2016; Nakada, 2019, and references therein). In Innocenti et al. (2019a), it is shown that dynamics of the memristor Murali-Lakshmanan-Chua circuit, equipped with an independent pulse programmed input source and simple comparator and hysteresis feedback blocks, can resemble some dynamical behaviors of cortical neurons. Finally, it is worth noting that HB techniques have been suitably employed for the analysis of neural oscillations (see e.g., Chen et al., 2008; Matsuoka, 2011 and references therein).

The purpose of this paper is to show how memristor circuits can be exploited for modeling some features of the neurons dynamics (see e.g., Izhikevich, 2000, 2007). The basic ideas are to exploit multistability, i.e., the fact that a memristor circuit contains infinitely many attractors (equilibria, limit cycles, …), and to employ a suitable pulse-programmed input for controlling multistability, i.e., for switching among the different attractors. Controlling multistability is currently a research field of general interest (see e.g., Pisarchik and Feudel, 2014 and references therein) and some contributions to the problem of steering the memristor circuit dynamics toward the attractors contained in one of the invariant manifolds have been given (Chen et al., 2018; Corinto and Forti, 2018; Corinto et al., 2019; Di Marco et al., 2019, 2020a,b). In particular, Di Marco et al. (2020b,a), Di Marco et al. (2021) have shown that the dynamics of a second order *R*, *L*, *C* circuit connected with a charge-controlled memristor can be steered, via a pulse programmed source, toward a pre-assigned invariant manifold where it converges toward the attractor contained in the manifold itself. In this paper, such a simple memristor circuit is used to mimic some aspects of the dynamics displayed by cortical neurons in response to an external stimulus. Section 2 describes the circuit and formulates the problem of interest. Specifically, the memristor charge should exhibit some typical neuron dynamics when the current source is suitably pulse-programmed. It is assumed that the pulses and their timing are generated by some given hardware mechanisms, whose design is not the object of the paper. Section 2 also illustrates the foliation property of the circuit state space in the input-less case, by characterizing the infinitely many invariant manifolds and the differential equations governing the dynamics onto them. Section 3 investigates the dynamics of the input-less circuit by showing that each manifold contains as attractor either a stable equilibrium point or a stable limit cycle. The limit cycles analysis is performed via the Describing Function (DF) method, a classical technique within the HB context. By exploiting the coexistence of stable equilibrium points and limit cycles and the knowledge of the first order periodic approximations, section 4 provides a procedure for the design of a pulse timing of the current source ensuring that the memristor charge mimics some dynamical features of the neuron response. The sought behavior of the memristor charge is obtained via a suitable concatenation of convergent and oscillatory behaviors, which are generated by switching between different attractors according to the designed pulse timing. The relation between the pulse timing parameters and the circuit parameters is also discussed. Some conclusions are finally drawn in section 5.

## 2. Problem Formulation and Preliminaries

**Figure 1:**
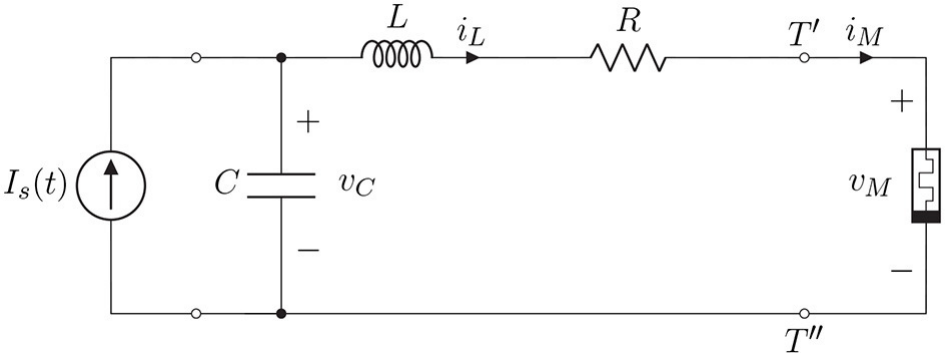
Charge-controlled memristor circuit with *R*, *L*,*C* components and independent current source *I*_*s*_.

The aim of the paper is to show that the coexistence of many different attractors in memristor circuits permits to mimic some features of the dynamics of cortical neurons. Specifically, we consider the simple circuit depicted in Figure 1 which contains a resistor *R*, an inductor *L*, a capacitor *C* and a non-linear charge-controlled memristor.

The capacitor voltage, the inductor current, the memristor voltage and current are denoted by *v*_*C*_, *i*_*L*_, *v*_*M*_, and *i*_*M*_, respectively. The circuit is subject to an independent current source *I*_*s*_, which is the input of the circuit. The charge-flux characteristic $\hat{\varphi }:\mathbb{R}\to \mathbb{R}$ of the memristor relating the charge *q*_*M*_ and the flux φ_*M*_ is assumed to have both a linear and a cubic term. Specifically,

(1) $$\begin{array}{l} \varphi_{M}=\hat{\varphi }\left(q_{M}\right)=-s_{0}q_{M}+\frac{s_{1}}{3}q_{M}^{3}\;, \end{array}$$

where the constant terms *s*_0_ and *s*_1_ satisfy

(2) $$\begin{array}{l} s_{0}>R\;,s_{1}>0\;. \end{array}$$

Throughout the paper, we assume that all the circuit parameters *R*, *L*, *C*, *s*_0_, *s*_1_ have normalized values. Also, we consider arbitrary units for the time.

**Figure 2:**
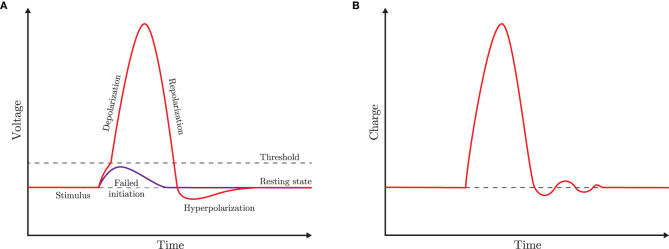
**(A)** Schematic of an ideal voltage-pulse of the cellular membrane (action potential). **(B)** Reference shape of the memristor charge-pulse considered in this paper. For the sake of simplicity, the depolarization is assumed to occur without distinction between the sub- and the super-threshold branches, and the hyperpolarization dynamics admits small ripples during the convergence to the resting state.

We want to show that the circuit is able to generate some characteristic dynamical behaviors of a neuron in response to a pulse stimulus, as the typical one depicted in Figure 2A. Specifically, the memristor charge *q*_*M*_ should display the time behavior of Figure 2B when the input source *I*_*s*_ provides a suitable stimulus.

It can be readily verified that the circuit dynamics is described by the following third-order system of differential equations

(3) $$\begin{array}{l} \{\begin{array}{c} Dv_{C}\left(t\right)\;=\frac{1}{C}\left(I_{s}\left(t\right)-i_{L}\right)\\ Dq_{M}\left(t\right)=i_{L}\\ Di_{L}\left(t\right)\;=\frac{1}{L}\left(v_{C}+\left(s_{0}-R\right)i_{L}-s_{1}q_{M}^{2}i_{L}\right) \end{array} \end{array}$$

where D is the time-derivative operator^1^ and *v*_*C*_, *q*_*M*_, and *i*_*L*_ are the state variables.

Let us consider the input-less case, i.e., *I*_*s*_ = 0. It has been shown (see e.g., Corinto and Forti, 2017) that memristor circuits enjoy the so-called foliation property, i.e., the memristor state space is decomposed into infinitely many invariant manifolds. The verification of this property for the considered circuit is readily obtained. Since *I*_*s*_ = 0, the first equation of Equation (3) can be rewritten equivalently as

(4) $$\begin{array}{l} Dv_{C}\left(t\right)+\frac{1}{C}i_{L}\left(t\right)=Dv_{C}\left(t\right)+\frac{1}{C}Dq_{M}\left(t\right)=\frac{1}{C}D\left(q_{M}\left(t\right)+Cv_{C}\left(t\right)\right)=0 \end{array}$$

which means that the quantity *q*_*M*_(*t*) + *Cv*_*C*_(*t*) is constant along the solutions of Equation (3) with initial conditions *v*_*C*_(*t*_0_), *q*_*M*_(*t*_0_), and *i*_*L*_(*t*_0_) at time *t* = *t*_0_, i.e.,

(5) $$\begin{array}{l} q_{M}\left(t\right)+Cv_{C}\left(t\right)=q_{M}\left(t_{0}\right)+Cv_{C}\left(t_{0}\right)\;\forall t\geq t_{0}\;. \end{array}$$

**Figure 3:**
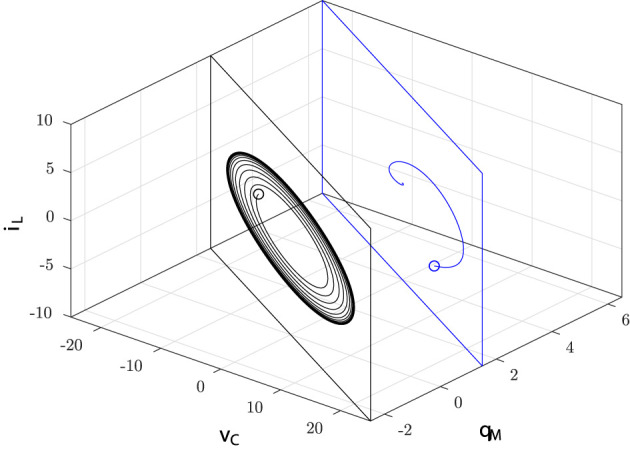
Invariant manifolds of the input-less circuit: the state space trajectory generated by the solution of Equation (3) with initial conditions *v*_*C*_(*t*_0_), *q*_*M*_(*t*_0_), *i*_*L*_(*t*_0_) (marked with ◦) at time *t* = *t*_0_ belongs to the manifold *M*(*Q*_0_) with *Q*_0_ = *q*_*M*_(*t*_0_) + *Cv*_*C*_(*t*_0_) for all *t* ≥ *t*_0_.

Hence, in the input-less case the state space of the memristor circuit is decomposed into infinitely many invariant manifolds of the form

(6) $$\begin{array}{l} M\left(Q_{0}\right)=\{\left(v_{C},q_{M},i_{L}\right):q_{M}\left(t\right)+Cv_{C}\left(t\right)=Q_{0}\;,\;\forall t\geq t_{0}\}, \end{array}$$

where *Q*_0_ is the index of the manifold whose value depends on the circuit initial conditions according to the relation *Q*_0_ = *q*_*M*_(*t*_0_) + *Cv*_*C*_(*t*_0_). Note that the invariant manifolds *M*(*Q*_0_) are planar surfaces parameterized by the manifold index *Q*_0_, as illustrated in Figure 3.

We have then established that in the input-less case the circuit dynamics is confined to lie onto one of the invariant manifolds. The differential equations governing the dynamics onto the invariant manifold *M*(*Q*_0_) can be readily singled out. Since the first equation of Equation (3) has been used to obtain *M*(*Q*_0_), it follows that the dynamics is characterized by the two remaining equations once *v*_*C*_(*t*) is replaced by $\frac{1}{C}\left(Q_{0}-q_{M}\left(t\right)\right)$. Hence, the dynamics onto *M*(*Q*_0_) obeys the second-order system of differential equations

(7) $$\begin{array}{l} \{\begin{array}{c} Dq_{M}\left(t\right)=i_{L}\left(t\right)\\ Di_{L}\left(t\right)\;=\frac{1}{LC}\left(Q_{0}-q_{M}\left(t\right)\right)+\frac{s_{0}-R}{L}i_{L}\left(t\right)-\frac{s_{1}}{L}q_{M}^{2}\left(t\right)i_{L}\left(t\right)\;. \end{array} \end{array}$$

Clearly, the complete dynamical behaviors of the input-less circuit can be obtained by collecting all the dynamics confined to lie onto each invariant manifold *M*(*Q*_0_). The dynamical analysis of Equation (7) for any value of the index manifold *Q*_0_ will be pursued in section 3. Note that *Q*_0_ can be seen as a parameter of Equation (7) which however depends on the circuit initial conditions and hence it has a different nature with respect to the circuit parameters *R*, *L*, *C*, *s*_0_, and *s*_1_.

## 3. Input-Less Memristor Circuit Dynamics

In this section, we summarize the properties of the dynamics onto *M*(*Q*_0_) by investigating system (7). Specifically, the equilibrium points and their stability features are dealt with in section 3.1, while the presence of limit cycles is considered in section 3.2.

### 3.1. Equilibrium Points

It readily follows that each invariant manifold *M*(*Q*_0_) has a unique equilibrium point at (*q*_*M*_, *i*_*L*_) = (*Q*_0_, 0). Taking into account (6), this implies that the circuit system (7) has an equilibrium point at (*v*_*C*_, *q*_*M*_, *i*_*L*_) = (0, *Q*_0_, 0) for any value of the index *Q*_0_. Hence, the *q*_*M*_-axis is a line of equilibrium points of the circuit.

To assess the stability properties of the equilibrium point of *M*(*Q*_0_), we compute the Jacobian *J*(*Q*_0_) of (7) at (*q*_*M*_, *i*_*L*_) = (*Q*_0_, 0), getting

(8) $$\begin{array}{l} J\left(Q_{0}\right)=\left(\begin{array}{cc} 0 & 1\\ -\frac{1}{LC} & \frac{s_{1}}{L}\left({\overset{-}{Q}}_{0}^{2}-Q_{0}^{2}\right) \end{array}\right)\;, \end{array}$$

where

(9) $$\begin{array}{l} {\overset{-}{Q}}_{0}=\sqrt{\frac{s_{0}-R}{s_{1}}}\;. \end{array}$$

The eigenvalues of *J*(*Q*_0_) have a negative real part if $|Q_{0}|>{\overset{-}{Q}}_{0}$ and a positive real part if $|Q_{0}|<{\overset{-}{Q}}_{0}$, while they are pure imaginary at $Q_{0}=\pm {\overset{-}{Q}}_{0}$. This implies that the equilibrium point at (*q*_*M*_, *i*_*L*_) = (*Q*_0_, 0) is ensured to be (locally) asymptotically stable if $|Q_{0}|>{\overset{-}{Q}}_{0}$ and unstable if $|Q_{0}|<{\overset{-}{Q}}_{0}$.

**Figure 4:**
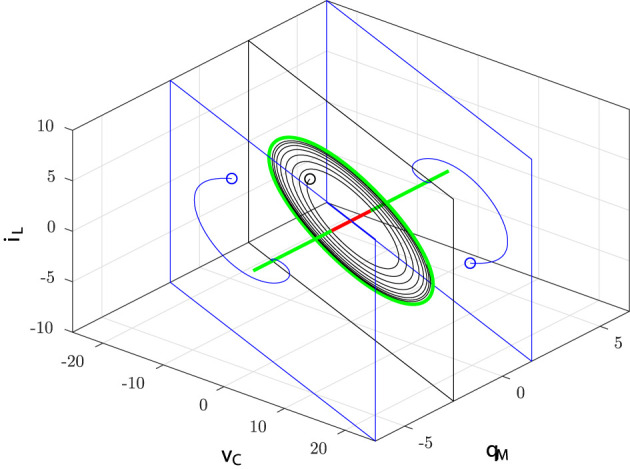
Stable (green half lines) and unstable (red segment) equilibrium points: the trajectories starting on the planes with $Q_{0}<-{\overset{-}{Q}}_{0}$ and $Q_{0}>{\overset{-}{Q}}_{0}$ converge toward the corresponding equilibrium points; the trajectory starting on the plane with $Q_{0}\in \left(-{\overset{-}{Q}}_{0},{\overset{-}{Q}}_{0}\right)$ converges toward the stable limit cycle (green).

Hence, the equilibrium point of each manifold *M*(*Q*_0_) with $|Q_{0}|>{\overset{-}{Q}}_{0}$ is an attractor of the input-less memristor circuit. Figure 4 summarizes the dynamical behavior around the equilibrium point of *M*(*Q*_0_).

### 3.2. Limit Cycles

**Figure 5:**
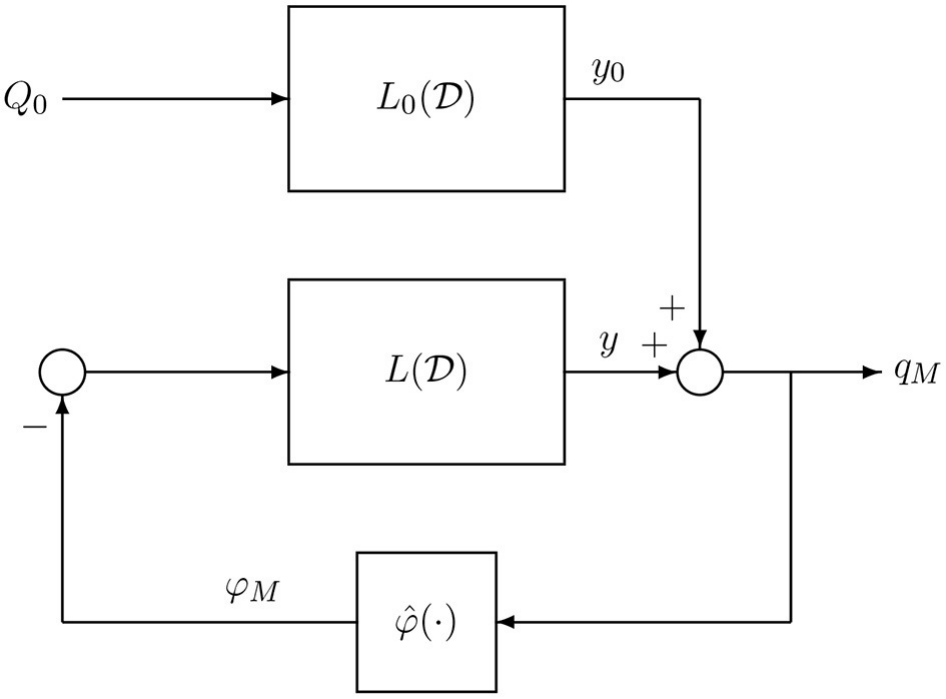
Equivalent input-output representation of system (7).

To investigate the presence of limit cycles on *M*(*Q*_0_) we resort to the DF method, a classical technique within the HB context. The DF method has been widely used for analyzing oscillatory behaviors in non-linear feedback control systems (see e.g., Gelb and Vander Velde, 1968; Atherton, 1975; Mees, 1981; Khalil, 2002). Within the HB setting, other techniques have been proposed to predict bifurcations of limit cycles and more complex dynamics (Genesio and Tesi, 1992; Piccardi, 1994; Tesi et al., 1996; Basso et al., 1997; Bonani and Gilli, 1999; Di Marco et al., 2003; Innocenti et al., 2010). The first step to apply the DF method is to show that system (7) admits the representation of Figure 5 whose dynamics is governed by the following input-output relation

(10) $$\begin{array}{l} q_{M}\left(t\right)=-L\left(D\right)\hat{\varphi }\left(q_{M}\left(t\right)\right)+L_{0}\left(D\right)Q_{0}\;, \end{array}$$

where L(D) and $L_{0}\left(D\right)$ are suitable rational functions.

It is worth observing that this representation has an internal feedback interconnection between the linear subsystem L(D) and the non-linear subsystem described by the memristor characteristic, while $L_{0}\left(D\right)$ is a feed-forward linear block driven by a constant input.

It can be readily verified that Equation (7) can be rewritten equivalently as a unique second order differential equation involving only *q*_*M*_. Since $i_{L}=Dq_{M}$ the second equation of Equation (7) becomes

(11) $$\begin{array}{l} D^{2}q_{M}\left(t\right)=\frac{1}{LC}\left(Q_{0}-q_{M}\left(t\right)\right)-\frac{R}{L}Dq_{M}\left(t\right)\\ \;-\frac{1}{L}\left(-s_{0}Dq_{M}\left(t\right)+s_{1}q_{M}^{2}\left(t\right)Dq_{M}\left(t\right)\right)\;. \end{array}$$

Taking into account that $-s_{0}Dq_{M}\left(t\right)+s_{1}q_{M}^{2}\left(t\right)Dq_{M}\left(t\right)=D{\hat{\varphi }}_{M}\left(t\right)$, the above differential equation can be rearranged in the following equivalent input-output form

(12) $$\begin{array}{l} q_{M}\left(t\right)=-\frac{\frac{1}{L}D}{D^{2}+\frac{R}{L}D+\frac{1}{LC}}\hat{\varphi }\left(q_{M}\left(t\right)\right)+\frac{\frac{1}{LC}}{D^{2}+\frac{R}{L}D+\frac{1}{LC}}Q_{0}\;. \end{array}$$

Hence, system (7) can be equivalently described via the input-output relation (10) with L(D) and $L_{0}\left(D\right)$ given by

(13) $$\begin{array}{l} L\left(D\right)=\frac{\frac{1}{L}D}{D^{2}+\frac{R}{L}D+\frac{1}{LC}}\;,\;L_{0}\left(D\right)=\frac{\frac{1}{LC}}{D^{2}+\frac{R}{L}D+\frac{1}{LC}}\;. \end{array}$$

It is worth observing that L(D) is exactly the equivalent admittance of the linear part of the circuit with *I*_*S*_(*t*) = 0, i.e., the one seen at the terminals *T*′ and *T*^′′^ in Figure 1 where the memristor is connected.

The DF method looks for a first-order approximation of a periodic output *q*_*M*_(*t*) of the system of Figure 5, i.e.,

(14) $$\begin{array}{l} q_{M}\left(t\right)=A+B\text{ cos }\omega t\;,\;B>0\;,\;\omega >0\;. \end{array}$$

The corresponding periodic output φ_*M*_(*t*) of period 2π/ω of the non-linear subsystem can be written as

(15) $$\begin{array}{l} \varphi_{M}\left(t\right)=\hat{\varphi }\left(A+B\text{ cos }\omega t\right)=\\ N_{0}\left(A,B\right)A+N_{1}\left(A,B\right)B\text{ cos }\omega t+\varphi_{h}\left(t\right), \end{array}$$

where *N*_0_(*A, B*) is the bias gain and *N*_1_(*A, B*) is first harmonic gain, which are known as describing function terms, while φ_*h*_(*t*) contains the higher order harmonics, i.e.,

(16) $$\begin{array}{l} \varphi_{h}\left(t\right)=\overset{+\infty }{\underset{h=2}{\sum }}\gamma_{h}\text{ cos }\left(h\omega t+\theta_{h}\right) \end{array}$$

with γ_*h*_ and θ_*h*_, *h* = 2, …, being suitable constants.

The final step of the DF method consists in first computing the periodic signal of period 2π/ω given by the sum of the outputs *y*(*t*) and *y*_0_(*t*) of the two linear subsystems driven by −φ_*M*_(*t*) and the constant signal *Q*_0_, respectively, then in equating the constant and the first order harmonic terms of the obtained periodic signal with the corresponding terms of *q*_*M*_(*t*), i.e., *A* and *B*. Taking into account that the constant and first order harmonic terms of φ_*M*_(*t*) can be rewritten in an exponential form as $N_{0}\left(A,B\right)Ae^{0t}$ and $N_{1}\left(A,B\right)B\left(e^{j\omega t}+e^{-j\omega t}\right)/2$, respectively, the periodic signal given by the sum of *y*(*t*) and *y*_0_(*t*) can be computed by exploiting superposition and the expression of the response of a linear system which has the same exponential form of the input^2^. The so computed periodic signal has the following expression

(17) $$\begin{array}{l} L\left(0\right)N_{0}\left(A,B\right)A+L_{0}\left(0\right)Q_{0}\\ +N_{1}\left(A,B\right)B\left(\frac{L\left(j\omega \right)e^{j\omega t}}{2}+\frac{L\left(-j\omega \right)e^{-j\omega t}}{2}\right)+y_{h}\left(t\right) \end{array}$$

where *y*_*h*_(*t*) contains the higher order harmonics generated by −φ_*h*_(*t*). Then, by equating the constant term in Equation (17) with that of *q*_*M*_(*t*), we get the real equation

(18) $$\begin{array}{l} A+L\left(0\right)N_{0}\left(A,B\right)A=L_{0}\left(0\right)Q_{0}\;, \end{array}$$

while by equating the first harmonic terms and taking into account that *B* cos ω*t* = *B*(*e*^*jωt*^ + *e*^−*jωt*^)/2 we get the complex equation

(19) $$\begin{array}{l} 1+L\left(j\omega \right)N_{1}\left(A,B\right)=0\;. \end{array}$$

In the HB approach, any first-order periodic signal (14) with *A*, *B*, and ω solving both Equations (18) and (19) is called a Predicted Limit Cycle (PLC) of the system of Figure 5. Also, it is expected that there exists a true limit cycle close to a PLC if the system of Figure 5 satisfies some conditions (see Atherton, 1975; Mees, 1981; Khalil, 2002 and references therein). These conditions basically rely on so-called filtering hypothesis which means that the non-linear subsystem does not generate large higher-order harmonics (i.e., φ_*h*_(*t*) is small) and the two linear subsystems are low-pass filters (i.e., the gains |*L*(*jhω*)|, *h* = 2, …, are smaller than |*L*(0)| and |*L*(*jω*)|). Hence, this hypothesis requires that *y*_*h*_(*t*) must be much smaller than the constant and first order harmonic terms of Equation (17), according to some quantitative measure. For instance, in Di Marco et al. (2018) the distortion index is used to this purpose.

Now, to apply the DF method to the system under consideration, we observe that from Equation (13) we get *L*(0) = 0 and *L*_0_(0) = 1, while from Equations (1) and (15) it can be verified that the gains *N*_0_(*A, B*) and *N*_1_(*A, B*) have the following expressions:

(20) $$\begin{array}{l} N_{0}\left(A,B\right)=-s_{0}+\frac{s_{1}}{3}\left(A^{2}+\frac{3}{2}B^{2}\right)\;, \end{array}$$

(21) $$\begin{array}{l} N_{1}\left(A,B\right)=-s_{0}+s_{1}\left(A^{2}+\frac{1}{4}B^{2}\right)\;. \end{array}$$

Then, the Equation (18) reduces to

(22) $$\begin{array}{l} A=Q_{0}\;, \end{array}$$

and the complex equation (19) boils down to

(23) $$\begin{array}{l} 1+\frac{\frac{1}{L}j\omega }{-\omega^{2}+\frac{R}{L}j\omega +\frac{1}{LC}}\left(-s_{0}+s_{1}\left(A^{2}+\frac{1}{4}B^{2}\right)\right)=0\;, \end{array}$$

which can equivalently be written by separating the real and imaginary parts as

(24) $$\begin{array}{l} R-s_{0}+s_{1}\left(A^{2}+\frac{1}{4}B^{2}\right)=0\;, \end{array}$$

(25) $$\begin{array}{l} -\omega^{2}+\frac{1}{LC}=0\;. \end{array}$$

Equations (22), (24), and (25) are then solved for *A* = Â = *Q*_0_, $\omega =\hat{\omega }=\frac{1}{\sqrt{LC}}$ and $B=\hat{B}$ with $\hat{B}$ such that

(26) $$\begin{array}{l} {\hat{B}}^{2}=4\left(\frac{s_{0}-R}{s_{1}}-Q_{0}^{2}\right)=4\left({\overset{-}{Q}}_{0}^{2}-Q_{0}^{2}\right)\;. \end{array}$$

Hence, for each index *Q*_0_ such that $|Q_{0}|<{\overset{-}{Q}}_{0}$ there exists a PLC of the following form

(27) $$\begin{array}{l} q_{M}\left(t\right)=Â+\hat{B}\text{ cos }\hat{\omega }t=Q_{0}+2\sqrt{{\overset{-}{Q^{2}}}_{0}-Q_{0}^{2}}\text{ cos }\frac{1}{\sqrt{LC}}t\;. \end{array}$$

This implies that each manifold *M*(*Q*_0_) contains a unique PLC for $|Q_{0}|<{\overset{-}{Q}}_{0}$. Since

(28) $$\begin{array}{l} i_{L}\left(t\right)=Dq_{M}\left(t\right)=-\hat{B}\hat{\omega }\text{ sin }\hat{\omega }t=-\frac{2}{\sqrt{LC}}\sqrt{{\overset{-}{Q^{2}}}_{0}-Q_{0}^{2}}\text{ sin }\frac{1}{\sqrt{LC}}t\;, \end{array}$$

the trajectory described by the PLC in the (*q*_*M*_, *i*_*L*_)-plane lies on the following ellipse

(29) $$\begin{array}{l} {\left(q_{M}-Q_{0}\right)}^{2}+LCi_{L}^{2}=4\left({\overset{-}{Q^{2}}}_{0}-Q_{0}^{2}\right)\;. \end{array}$$

Note that the ellipse is centered at the equilibrium point (*Q*_0_, 0) and its size is maximum at *Q*_0_ = 0 and decreases to zero as the index *Q*_0_ tends to ${\overset{-}{Q}}_{0}$, thus collapsing to the equilibrium point. Moreover, since for $|Q_{0}|<{\overset{-}{Q}}_{0}$ the equilibrium point is unstable and taking into account that the system is two dimensional, we can conclude that the PLC is stable. In this regard, we mention that PLC stability can be assessed also via approximate HB tools, such as the Loeb criterion (see e.g., Atherton, 1975; Tesi et al., 1996).

**Figure 6:**
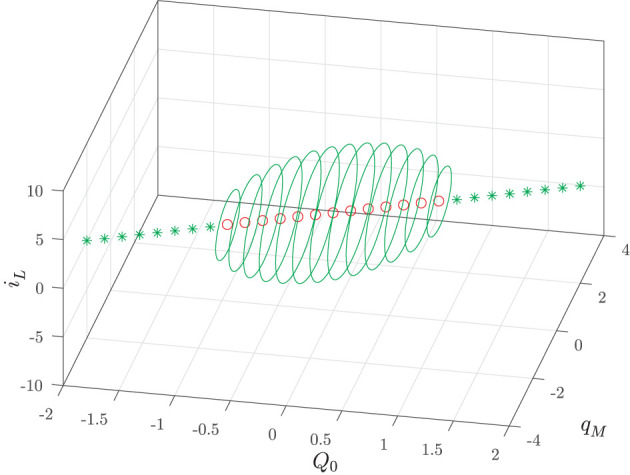
Input-less circuit attractors: stable (green ⋆) equilibrium points and stable PLCs (solid green) given by Equations (27) and (28) as a function of the manifold index *Q*_0_. The red circles denote the unstable equilibrium points.

Summing up, we have shown that in the input-less memristor circuit there coexist infinitely many attractors: a stable equilibrium point for each value of the manifold index *Q*_0_ such that $|Q_{0}|>{\overset{-}{Q}}_{0}$ and a stable PLC for each value of the index *Q*_0_ such that $|Q_{0}|<{\overset{-}{Q}}_{0}$. Figure 6 illustrates this multistability scenario for the following normalized values of the circuit parameters

(30) $$\begin{array}{l} R=0.4\;,\;C=0.1\;,\;L=1.5\;,\;s_{0}=0.7\;,\;s_{1}=0.3\;, \end{array}$$

from which it follows that ${\overset{-}{Q}}_{0}=1$.

**Figure 7:**
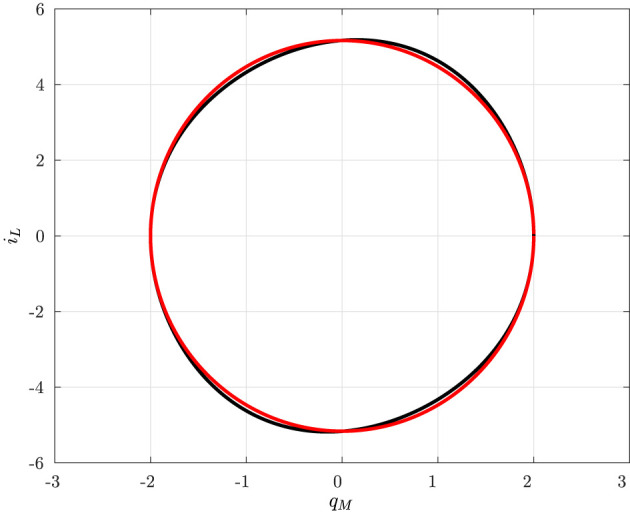
True limit cycle (solid dark) and PLC (solid red) in the (*q*_*M*_, *i*_*L*_)-plane for *Q*_0_ = 0.

In particular, it turns out that at $Q_{0}=\pm {\overset{-}{Q}}_{0}=\pm 1$ a typical behavior of (supercritical) Hopf bifurcation is observed. However, since it is obtained by varying the index *Q*_0_ and thus the circuit initial conditions (for fixed circuit parameters), it may be referred to as a bifurcation without parameters (Corinto and Forti, 2017; Di Marco et al., 2018; Innocenti et al., 2019b; Ascoli et al., 2020a). As already discussed, it is expected that there exists a true limit cycle close to a PLC. Indeed, a more refined numerical analysis shows that for each |*Q*_0_| < 1 the corresponding invariant manifold *M*(*Q*_0_) has a unique limit cycle which attracts all the (non-trivial) trajectories. Moreover, the limit cycle is well-approximated by the PLC, as shown in Figure 7 for *Q*_0_ = 0.

**Figure 8:**
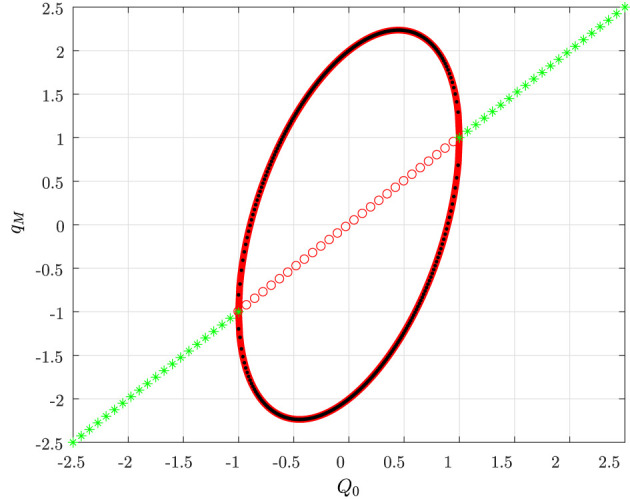
Stable (green ⋆) and unstable (red ◦) equilibrium points; maximum (32) and minimum (31) of the PLCs (solid red) as a function of *Q*_0_; maximum and minimum values of the numerically computed limit cycles (dotted dark points).

A quantitative comparison between the PLCs and the true limit cycles is provided by Figure 8 where the maximum and the minimum of both the true periodic solution *q*_*M*_(*t*) and the PLC in Equation (27) are depicted as a function of the index *Q*_0_.

It can be readily checked that the minimum and the maximum of the first-order approximation are given by

(31) $$\begin{array}{l} \underset{t}{\min}q_{M}\left(t\right)=Q_{0}-2\sqrt{{\overset{-}{Q^{2}}}_{0}-Q_{0}^{2}} \end{array}$$

and

(32) $$\begin{array}{l} \underset{t}{\max}q_{M}\left(t\right)=Q_{0}+2\sqrt{{\overset{-}{Q^{2}}}_{0}-Q_{0}^{2}}\;, \end{array}$$

respectively. We finally observe that similar results can be derived also for values of the circuit parameters different from those in Equation (30).

## 4. Modeling Neuron Dynamics via the Controlled Circuit

Section 3 makes it clear that in the input-less case the memristor circuit displays infinitely many attractors. Specifically, each planar invariant manifold *M*(*Q*_0_) contains either a unique stable equilibrium point or a stable limit cycle. Moreover, an approximation of the limit cycle belonging to *M*(*Q*_0_), with *Q*_0_ such that $|Q_{0}|<{\overset{-}{Q}}_{0}$, is explicitly obtained in terms of the PLC (27).

In this section it will be shown how the coexistence of infinitely many stable equilibrium points and limit cycles, together with the knowledge of their dependence on the manifold index *Q*_0_, which in the case of limit cycles is given in an approximate way by Equation (27), can be suitably exploited to make the memristor charge *q*_*M*_ responding as in Figure 2B to a pulse shaped input source *I*_*s*_. To proceed, we consider the circuit state equations (3) by replacing *v*_*C*_ with the following state variable

(33) $$\begin{array}{l} Q≐q_{M}+Cv_{C}\;. \end{array}$$

It turns out that (3) reduces to the third-order system

(34) $$\begin{array}{l} \{\begin{array}{c} DQ\left(t\right)\;=I_{s}\left(t\right)\\ Dq_{M}\left(t\right)=i_{L}\\ Di_{L}\left(t\right)\;=\frac{1}{LC}\left(Q-q_{M}\right)+\frac{s_{0}-R}{L}i_{L}-\frac{s_{1}}{L}q_{M}^{2}i_{L} \end{array} \end{array}$$

where *Q*, *q*_*M*_, and *i*_*L*_ are the new state variables. Suppose that at time *t* = *t*_*i*_ ≥ *t*_0_ the current source *I*_*s*_(*t*) displays a pulse of area Λ and width Δ > 0, i.e., *I*_*s*_(*t*) is equal to zero for *t* ∈ [*t*_0_, *t*_*i*_] and *t* ∈ [*t*_*i*_ + Δ, +∞) and such that

(35) $$\begin{array}{l} \int_{t_{i}}^{t_{i}+\Delta }I_{s}\left(t\right)dt=\Lambda \;. \end{array}$$

From the first equation of Equation (34) it follows that

(36) $$\begin{array}{l} Q\left(t\right)=Q_{0}^{\left(i\right)}+\int_{t_{i}}^{t}I_{s}\left(\sigma \right)d\sigma \;, \end{array}$$

which implies $Q\left(t\right)=Q_{0}^{\left(i\right)}$ for *t* ∈ [*t*_0_, *t*_*i*_] and $Q\left(t\right)=Q_{0}^{\left(i\right)}+\Lambda$ for *t* ∈ [*t*_*i*_ + Δ, +∞). For instance, in the case of a rectangular pulse we have $Q\left(t\right)=Q_{0}^{\left(i\right)}+\left(A/\Delta \right)\left(t-t_{i}\right)$.

By comparing the second and third equations of Equation (34) with those of Equation (7), it follows that the circuit dynamics lies onto $\text{M}\left(Q_{0}^{\left(i\right)}\right)$ for *t* ∈ [*t*_0_, *t*_*i*_], it moves toward $\text{M}\left(Q_{0}^{\left(i\right)}+\Lambda \right)$ during the interval [*t*_*i*_, *t*_*i*_ + Δ], it reaches $\text{M}\left(Q_{0}^{\left(i\right)}+\Lambda \right)$ at *t* = *t*_*i*_ + Δ, where it remains for all *t* ≥ *t*_*i*_ + Δ. Hence, the circuit has the property that pulse shaped current sources *I*_*s*_ make the dynamics moving from an initial manifold to any other one within a given time interval, by suitably choosing the pulse area and width.

**Figure 9:**
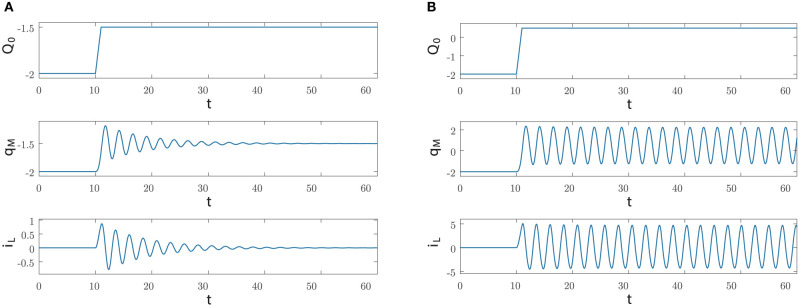
Dynamics generated by applying at *t*_*i*_ = 10 a rectangular pulse of area Λ and width Δ = 1 via the current source *I*_*s*_; the circuit parameters are as in Equation (30) and the initial conditions are *q*_*M*_(0) = −2 and *I*_*L*_(0) = 0, while *Q*(0) = −2. **(A)** For Λ = 0.5, the index *Q*(*t*) and the state variables *q*_*M*_(*t*), *i*_*L*_(*t*) converge to *Q*_0_ = −1.5 and to the equilibrium point (−1.5, 0), respectively. **(B)** For Λ = 2.5, *Q*(*t*) converges to *Q*_0_ = 0.5 and the state variables *q*_*M*_(*t*), *i*_*L*_(*t*) converge to the limit cycle belonging to the manifold *M*(0.5).

We are interested in the case where the manifold $\text{M}\left(Q_{0}^{\left(i\right)}\right)$ has a stable equilibrium point and in particular the index $Q_{0}^{\left(i\right)}$ is negative and hence $Q_{0}^{\left(i\right)}<-{\overset{-}{Q}}_{0}$. Moreover, we assume that at *t* = *t*_*i*_ the circuit state is at the equilibrium point, which means that $\left(Q\left(t_{i}\right),q_{M}\left(t_{i}\right),i_{L}\left(t_{i}\right)\right)=\left(Q_{0}^{\left(i\right)},Q_{0}^{\left(i\right)},0\right)$. Let us now investigate the dynamics induced by a rectangular pulse of positive area Λ and width Δ on the circuit dynamics. It turns out that the final manifold $\text{M}\left(Q_{0}^{\left(i\right)}+\Lambda \right)$ still contains a stable equilibrium point if $\Lambda <|Q_{0}^{\left(i\right)}|-{\overset{-}{Q}}_{0}$, while it contains a stable limit cycle if $|Q_{0}^{\left(i\right)}|-{\overset{-}{Q}}_{0}<\Lambda <|Q_{0}^{\left(i\right)}|+{\overset{-}{Q}}_{0}$ and again a stable equilibrium point if $\Lambda >|Q_{0}^{\left(i\right)}|+{\overset{-}{Q}}_{0}$. Figure 9 illustrates the first two possible behaviors in the case of a rectangular pulse of height *A*/Δ with the circuit parameters as in (30).

Specifically, after reaching $\text{M}\left(Q_{0}^{\left(i\right)}+\Lambda \right)$ at time *t* = *t*_*i*_ + Δ at the point (*Q*(*t*_*i*_ + Δ), *q*_*M*_(*t*_*i*_ + Δ), *i*_*L*_(*t*_*i*_ + Δ)) with $Q\left(t_{i}+\Delta \right)=Q_{0}^{\left(i\right)}+\Lambda$, the dynamics converges toward the attractor contained in the manifold, an equilibrium point in Figure 9A and a limit cycle in Figure 9B.

Clearly, the length of the transient behavior toward the attractor depends on the values of *q*_*M*_(*t*_*i*_ + Δ) and *i*_*L*_(*t*_*i*_ + Δ). The farther away is the point (*q*_*M*_(*t*_*i*_ + Δ), *i*_*L*_(*t*_*i*_ + Δ)) from the attractor lying onto $\text{M}\left(Q_{0}^{\left(i\right)}+\Lambda \right)$, the longer is the transient. Clearly, if (*q*_*M*_(*t*_*i*_ + Δ), *i*_*L*_(*t*_*i*_ + Δ)) belongs to the attractor we have no transient behavior. The values of *q*_*M*_(*t*_*i*_ + Δ) and *i*_*L*_(*t*_*i*_ + Δ) cannot be computed explicitly since this would require to integrate the second and third equations of (34) in the interval [*t*_*i*_, *t*_*i*_ + Δ] with the initial condition $\left(q_{M}\left(t_{i}\right),i_{L}\left(t_{i}\right)\right)=\left(Q_{0}^{\left(i\right)},0\right)$ and $Q\left(t\right)=Q_{0}^{\left(i\right)}+\Lambda \left(t-t_{i}\right)/\Delta$. However, by employing a Taylor series expansion for *q*_*M*_(*t*) and *i*_*L*_(*t*) for *t* ∈ [*t*_*i*_, *t*_*i*_ + Δ], it turns out that

(37) $$\begin{array}{l} q_{M}\left(t_{i}+\Delta \right)=q_{M}\left(t_{i}\right)+\alpha_{1}\Delta +\alpha_{2}\Delta^{2}+\ldots =Q_{0}^{\left(i\right)}+O\left(\Delta \right)\\ i_{L}\left(t_{i}+\Delta \right)=i_{L}\left(t_{i}\right)+\beta_{1}\Delta +\beta_{2}\Delta^{2}\ldots \;=\beta_{1}\Delta +O\left(\Delta^{2}\right)\;, \end{array}$$

where α_*i*_ and β_*i*_, *i* = 1, 2 are constants. It is interesting to note that if the width Δ of the pulse is small, then for *t* ∈ [*t*_*i*_, *t*_*i*_ + Δ] the charge *q*_*M*_ remains almost constant, while *i*_*L*_ varies from zero to a quantity proportional to Δ.

Summing up, for a suitable choice of the pulse area *A* and for sufficiently small width Δ, the pulse current *I*_*s*_ is able to steer, within the interval [*t*_*i*_, *t*_*i*_ + Δ], the dynamics from the stable equilibrium point of $\text{M}\left(Q_{0}^{\left(i\right)}\right)$ to the manifold $\text{M}\left(Q_{0}^{\left(i\right)}+A\right)$ containing a stable limit cycle. Moreover, during the time interval the charge *q*_*M*_ is almost constant and the current *i*_*L*_ remains close to zero.

**Figure 10:**
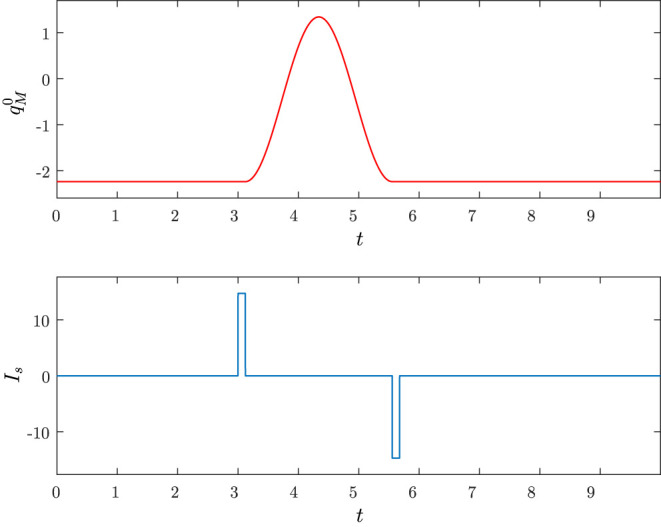
**Top**: predicted behavior $q_{M}^{0}\left(t\right)$ of the memristor charge. **Bottom**: timing of the current pulses.

We are now ready to show how it is possible to make the charge *q*_*M*_ display a behavior similar to that of Figure 2B in response to a pulse shaped input source *I*_*s*_. Here, we are more interested in the dynamic response of the neuron than in its set and reset mechanisms. For these mechanisms we simply adopt the pulse timing of Figure 10 which is assumed to be generated via suitable logic gates.

At time *t* = *t*_*i*_ the hardware detects a stimulus and generates a current pulse *I*_*s*_ with area *A* and width Δ (set mechanism), which moves the dynamics away from the stable equilibrium point (resting point) in $\text{M}\left(Q_{0}^{\left(i\right)}\right)$; at time *t* = *t*_*i*_ + Δ the dynamics reaches $\text{M}\left(Q_{0}^{\left(i\right)}+\Lambda \right)$ and the memristor charge *q*_*M*_ starts displaying a behavior similar to that reported in Figure 2B; at time *t* = *t*_*i*_ + Δ + *T* an inverse current pulse is generated in order to make the dynamics come back to the stable equilibrium point in $\text{M}\left(Q_{0}^{\left(i\right)}\right)$ (reset mechanism).

Hence, the problem is how the parameters $Q_{0}^{\left(i\right)}$, Λ, Δ, and *T* should be designed in order to obtain the sought behavior of *q*_*M*_. Let Δ be chosen enough small to ensure, as discussed above, that in [*t*_*i*_, *t*_*i*_ + Δ] the charge *q*_*M*_ is almost constant and the current *i*_*L*_ remains close to zero. Choosing a small Δ implies that when at *t* = *t*_*i*_ + Δ the dynamics reaches the manifold $\text{M}\left(Q_{0}^{\left(i\right)}+\Lambda \right)$ we have that *q*_*M*_ and *i*_*L*_ are quite close to $Q_{0}^{\left(i\right)}$ and zero, respectively. This manifold contains a stable limit cycle which can be in general computed only numerically. However, in section 3 it has been shown that the limit cycle can be approximated by a PLC. Clearly, the current expression of the PLC on $\text{M}\left(Q_{0}^{\left(i\right)}+\Lambda \right)$ is obtained by putting $Q_{0}=Q_{0}^{\left(i\right)}+\Lambda$ in Equation (27).

Now, observe that the minimum and the maximum values of *q*_*M*_, expressed in Equations (31) and (32), respectively, are achieved once *i*_*L*_ is equal to zero. Hence, since for sufficiently small Δ we have that $\left(q_{M}\left(t_{i}+\Delta \right),i_{L}\left(t_{i}+\Delta \right)\right)\approx \left(Q^{\left(i\right)},0\right)$, it is possible to set (*q*_*M*_(*t*_*i*_ + Δ), *i*_*L*_(*t*_*i*_ + Δ)) as the point of the manifold $\text{M}\left(Q_{0}^{\left(i\right)}+\Lambda \right)$ where the PLC has its minimum value (31). Consequently, for *t* ∈ [*t*_*i*_ + Δ, *t*_*i*_ + Δ + *T*] with *T* being the PLC period, the PLC provides the typical harmonic oscillator over one period, i.e., it evolves from the minimum to the maximum coming back again to the minimum. At the end of the period, the reset mechanism generates a current pulse of area −*A* and width Δ, which makes the dynamics come back to the stable equilibrium point of $\text{M}\left(Q_{0}^{\left(i\right)}\right)$. Hence, the resulting behavior of the memristor charge $q_{M}^{0}\left(t\right)$ can be expressed as

(38) $$\begin{array}{l} q_{M}^{0}(t)=\{\begin{array}{ll} Q_{0}^{\left(i\right)} & t\in [t_{0},t_{i}+\Delta ]\\ Q_{0}^{\left(i\right)}+\Lambda +2\sqrt{{\bar{Q^{2}}}_{0}-{\left(Q_{0}^{\left(i\right)}+\Lambda \right)}^{2}}\cos\frac{1}{\sqrt{LC}}(t-t_{i}-\Delta )+\pi ) & t\in (t_{i}+\Delta ,t_{i}+\Delta +T)\\ Q_{0}^{\left(i\right)} & t\in (t_{i}+\Delta +T,+\infty ) \end{array} \end{array}$$

and is depicted in Figure 10. Note that the parameter *T* has been chosen as the period of the PLC, i.e.:

(39) $$\begin{array}{l} T=2\pi \sqrt{LC}\;. \end{array}$$

Hereafter, we denote by $i_{L}^{0}\left(t\right)$ the time-derivative of $q_{M}^{0}\left(t\right)$, i.e.:

(40) $$\begin{array}{l} i_{L}^{0}\left(t\right)=Dq_{M}^{0}\left(t\right)\;\forall t\in \left[t_{0},+\infty \right)\;. \end{array}$$

Now, to set (*q*_*M*_(*t*_*i*_ + Δ), *i*_*L*_(*t*_*i*_+Δ)) as the point of the manifold $\text{M}\left(Q_{0}^{\left(i\right)}+\Lambda \right)$ where the PLC has its minimum value (31), the following condition must be satisfied

(41) $$\begin{array}{l} Q_{0}^{\left(i\right)}=Q_{0}-2\sqrt{{\overset{-}{Q}}_{0}^{2}-Q_{0}^{2}} \end{array}$$

with $Q_{0}=Q_{0}^{\left(i\right)}+\Lambda$. It is not difficult to verify that there are many solutions $Q_{\left(i\right)}^{0}$ and *Q*_0_ of (41) such that $Q_{0}^{\left(i\right)}<-{\overset{-}{Q}}_{0}$ and $|Q_{0}|<{\overset{-}{Q}}_{0}$. We are interested in the one with the minimum value of $Q_{0}^{\left(i\right)}$ because this choice ensures that the stable equilibrium point on the initial manifold $\text{M}\left(Q_{0}^{\left(i\right)}\right)$ is at the maximum distance from the invariant manifold $\text{M}\left({\overset{-}{Q}}_{0}\right)$ where the Hopf bifurcation happens, thus guaranteeing a larger robustness margin against spurious pulses of small area. The minimum value of $Q_{0}^{\left(i\right)}$ can be computed in closed form by minimizing the right hand side of Equation (41). It turns out that

(42) $$\begin{array}{l} Q_{0}≐Q_{0}^{*}=-\frac{1}{\sqrt{5}}{\overset{-}{Q}}_{0}\;, \end{array}$$

and since

$$\begin{array}{l} Q_{0}^{*}-2\sqrt{{\overset{-}{Q}}_{0}^{2}-{\left(Q_{0}^{*}\right)}^{2}}=-\sqrt{5}{\overset{-}{Q}}_{0}\;, \end{array}$$

from Equations (41) and (9) we have

(43) $$\begin{array}{l} Q_{0}^{\left(i\right)}=-\sqrt{5}\sqrt{\frac{s_{0}-R}{s_{1}}}\;. \end{array}$$

Moreover, since *Q*_0_ in Equation (41) stands for $Q_{0}^{\left(i\right)}+A$, from Equation (42) we finally get

(44) $$\begin{array}{l} \Lambda =\frac{4}{\sqrt{5}}\sqrt{\frac{s_{0}-R}{s_{1}}}\;. \end{array}$$

Finally, the pulse width Δ should be practically chosen to be some percent of the period *T*. Hence, we can select it according to the relation

(45) $$\begin{array}{l} \Delta =2\sigma \pi \sqrt{LC}\;, \end{array}$$

where σ lies in the interval [0.01, 0.05].

Summing up, to obtain $q_{M}^{0}\left(t\right)$ in Equation (38), the parameters *T*, $Q_{0}^{\left(i\right)}$, Λ, and Δ can be designed according to the corresponding expressions given in Equations (39), (43), (44), and (45), respectively. Notably, these relations depend explicitly on the circuit parameters *R*, *L*, *C*, *s*_0_, and *s*_1_. Hence, by suitably adapting these parameters it is possible to vary the features of $q_{M}^{0}\left(t\right)$.

On the other hand, the formulas in Equations (39), (43), (44), and (45) have been derived under two approximations. The first one concerns the assumption that at time *t* = *t*_*i*_ + Δ the values of *q*_*M*_ and *i*_*L*_ are quite close to $Q_{0}^{\left(i\right)}$ and zero, respectively. However, according to Equation (37) by lowering the value of the pulse width this assumption can be suitably satisfied. The second approximation is that the formulas have been obtained by using the PLCs instead of the true limit cycles. Indeed, as shown in section 3.2, the true limit cycles contain higher order harmonics terms which can generate some distortion in both the period and the minimum and maximum amplitudes over the period, with respect to those analytically computed for the PLCs. However, these higher order harmonics can be filtered out by suitably adjusting the circuit parameters. Moreover, the fact that the input-less memristor circuit contains a bundle of limit cycles makes the formulas, and hence the parameter design procedure, sufficiently robust with respect to the use of PLC in their derivation. Said another way, by slightly varying the parameters *T*, $Q_{0}^{\left(i\right)}$, *A*, and Δ from the nominal values provided by Equations (39), (43), (44), and (45), respectively, the distortion effect could be reduced.

To illustrate the design procedure let us consider the circuit parameters in Equation (30). From Equations (39), (43), and (44), we have that

(46) $$\begin{array}{l} T=2.4334\;,\;Q_{0}^{\left(i\right)}=-2.2361\;,\;\Lambda =1.7889\;, \end{array}$$

and choosing σ = 0.02 in Equation (45) we obtain

(47) $$\begin{array}{l} \Delta =0.0487\;. \end{array}$$

**Figure 11:**
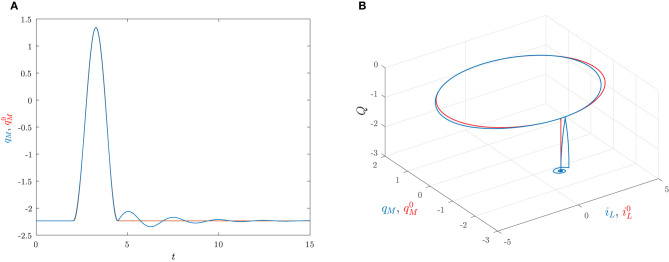
The set pulse is applied at *t*_*i*_ = 2 with area *A* = 1.7889 and width Δ = 0.0487, the reset pulse is applied at *t*_*i*_ + Δ + *T* = 4.4821 with area Λ = −1.7889 and width Δ = 0.0487. The initial conditions are *Q*(0) = −2.2361, *q*_*M*_(0) = −2.2361 and *i*_*L*_(0) = 0. **(A)** Time behaviors of *q*_*M*_ (blu) and $q_{M}^{0}$ (red). **(B)** Trajectories in the state space generated by (*Q*(*t*), *q*_*M*_(*t*), *i*_*L*_(*t*)) (blu) and $\left(Q\left(t\right),q_{M}^{0}\left(t\right),i_{L}^{0}\left(t\right)\right)$ (red).

The corresponding predicted behavior $q_{M}^{0}\left(t\right)$ in Equation (38) is reported Figure 11A together with the true behavior of *q*_*M*_(*t*) which is obtained by solving numerically (34). The state space trajectories corresponding to the true solution (*Q*(*t*), *q*_*M*_(*t*), *i*_*L*_(*t*)) of the circuit and the predicted solution $\left(Q\left(t\right),q_{M}^{0}\left(t\right),i_{L}^{0}\left(t\right)\right)$) are depicted in Figure 11B.

Note that the charge *q*_*M*_(*t*) has a dynamical behavior that is quite similar to the one depicted in Figure 2B, thus clearly showing that the memristor circuit can be used for mimicking some features of neuron dynamics.

**Figure 12:**
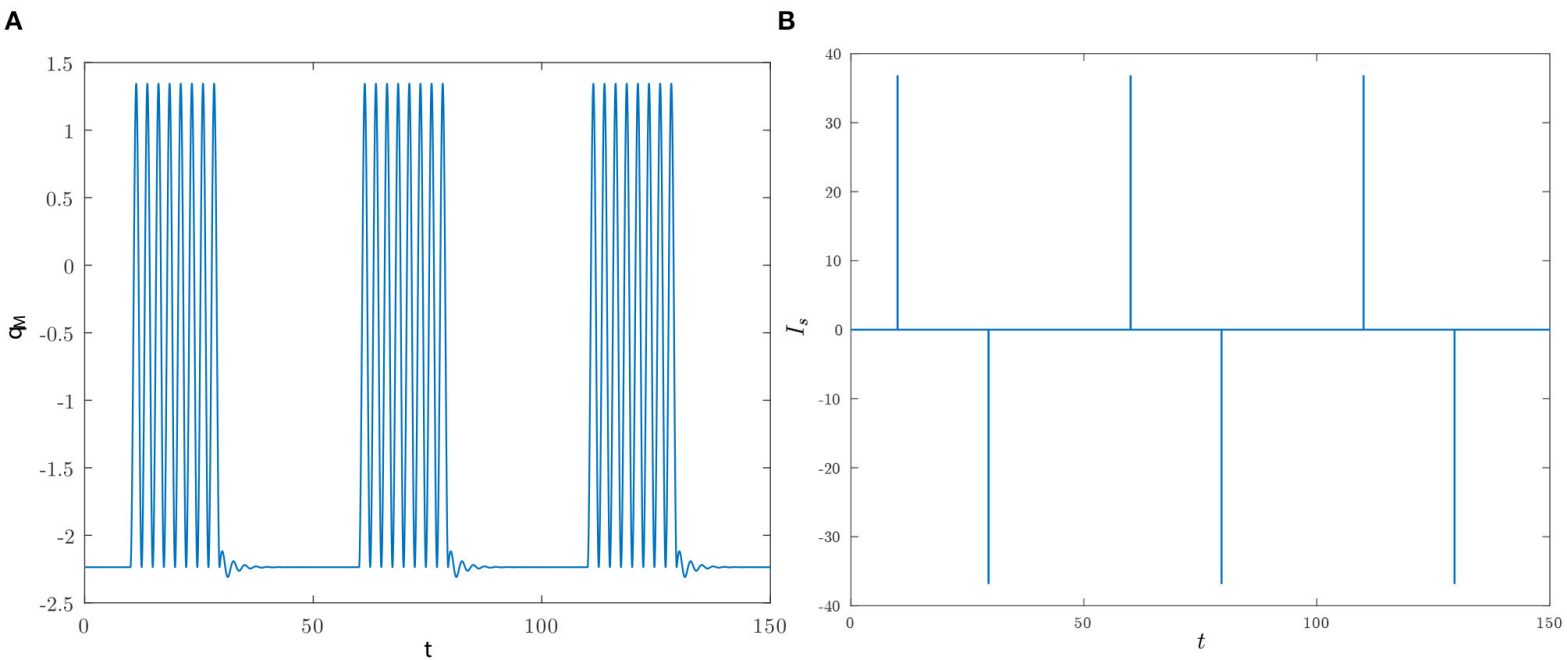
The initial conditions are as in Figure 11, the set pulses are activated at *t*_*i*_ ∈ {10, 60, 110} and the reset pulses are applied 19.5164 time units later. **(A)** Time behavior of *q*_*M*_ for *n* = 8. **(B)** Time behavior of *I*_*s*_.

This dynamical similarity is confirmed also in other scenarios. For instance, suppose that in the pulse timing the reset pulse is activated at *t* = *t*_*i*_ + Δ + *nT*, with *n* being a positive integer. Clearly, in this case $q_{M}^{0}\left(t\right)$ completes *n* periods within the interval *t*_*i*_ + Δ, *t*_*i*_ + Δ + *nT*, and, therefore, *q*_*M*_(*t*) is expected to generate a burst of *n* spikes. Figure 12 provides the numerically computed behavior of *q*_*M*_(*t*) for *n* = 8 over a time frame covering three bursts.

## 5. Conclusions

In this paper, a memristor circuit composed of a resistor, an inductor, a capacitor, an ideal charge-controlled memristor and an independent current source as input is considered. It is first shown that in the input-less case the circuit enjoys the foliation property of the state space, i.e., it contains infinitely many planar invariant manifolds which are parameterized by a scalar index depending on the circuit initial conditions. Each manifold contains an attractor which can be either a stable equilibrium point or a stable limit cycle, depending on the value of the manifold index. Moreover, a first-order periodic approximation is obtained in an analytic way for each limit cycle via the Describing Function (DF) technique, a classical tool within the Harmonic Balance (HB) context.

Then, it is shown that the memristor charge can mimic a simplified model of a neuron response when an external independent pulse-programmed current source is introduced in the circuit. Specifically, the sought dynamics of the memristor charge is generated via the concatenation of convergent and oscillatory behaviors, which are obtained by switching between stable equilibrium points and limit cycles via a suitable design of the pulse timing of the current source. Some relationships between the pulse and the circuit parameters are also devised exploiting the knowledge of the first-order periodic approximation of the limit cycles.

## Data Availability Statement

The raw data supporting the conclusions of this article will be made available, without undue reservation, on request to the corresponding author with appropriate motivation.

## Author Contributions

All authors contributed to the conception and design of the study, wrote the manuscript, read, and approved the submitted version.

## Conflict of Interest

The authors declare that the research was conducted in the absence of any commercial or financial relationships that could be construed as a potential conflict of interest.

## Footnotes

^1^Throughout the paper, $Df\left(\overset{-}{t}\right)$ denotes the derivative of the function *f*(*t*) at $t=\overset{-}{t}$.

^2^Consider a linear time-invariant finite-dimensional causal system described by the input-output relation y(t)=L(D)u(t) where the operator L(D) has the following general form

$$L\left(D\right)=\frac{b_{n-1}D^{n-1}+\ldots +b_{1}D+b_{0}}{D^{n}+a_{n-1}D^{n-1}+\ldots +a_{1}D+a_{0}}$$

with *a*_*i*_, *b*_*i*_, *i* = 0, …, *n* − 1 being constants. Let *u*(*t*) be an exponential signal, i.e., *u*(*t*) = *Ce*^σ*t*^, *C* ∈ ℝ, σ ∈ ℂ, and assume that *L*(σ) is finite, i.e., *L*(σ) ≠ ∞. Then, for suitable initial conditions of the system, we have *y*(*t*) = *CL*(σ)*e*^σ*t*^, i.e., the output of the system has the same exponential form of the input.
